# Reviewed article: Nery DP, Andrade ACS, Rocha DS, Barbosa MCR, Gomes RS, Bezerra VM. Trend in neonatal mortality and preventable neonatal deaths: a time series analysis, Bahia, 2010-2020. Epidemiol Serv Saude.2025:34;e20240651

**DOI:** 10.1590/S2237-96222025v34e20240651.b

**Published:** 2025-09-29

**Authors:** Betine Pinto Moehlecke Iser

**Affiliations:** 1 Universidade do Sul de Santa Catarina, Tubarão, SC, Brazil

## First round comments

Caros autores

Parabéns pelo seu trabalho. O estudo apresenta as taxas de mortalidade neonatal na Bahia e apresenta dados relevantes ao SUS, sendo adequado ao escopo da RESS. O texto em geral está bem escrito e fundamentado. A análise da mortalidade neonatal a partir da evitabilidade das causas de óbito é um diferencial deste estudo e permite a orientação de estratégias de mitigação, trazendo maior aplicabilidade aos serviços de saúde. 

Acredito que este ponto poderia ser melhor explorado na discussão, de forma a direcionar politicas públicas específicas.

Outro ponto importante refere-se a possível influência de 2020, ano no qual enfrentamos a pandemia de COVID-19, na série histórica apresentada. Os autores chegaram a avaliar se a exclusão de 2020 da análise traria diferença aos dados apresentados? Pelos dados da tabela 1, parece que em 2020 houve redução da taxa de mortalidade neonatal precoce... Estaria este ano em específico influenciando a tendência como um todo? Solicita-se incluir considerações sobre esta questão também na discussão dos principais resultados.

Os nossos revisores fizeram considerações importantes para a melhoria do seu manuscrito. Avalie com atenção a cada ponto para reapresentá-lo. Nos métodos, atente-se ao indicado pelos revisores, especialmente quanto a constante utilizada no cálculo (10 mil ao invés de 1.000), e indique a forma de cálculo das taxas médias apresentadas. A forma de apresentação dos resultados pode ser aprimorada, e a discussão pode ser aprofundada, conforme detalhamento apresentado por cada revisor.

Acrescento ainda algumas questões menores no texto:

Resumo

Podem ser retiradas expressões como “Neste estudo se objetivou...” (linha 9) e “Realizou-se um” estudo de série temporal... (linha 11)

Linha 12: o n=26.661 refere-se ao método ou seria o primeiro resultado do estudo, como apresentado no decorrer do manuscrito (página 3/18, linha 40)?

Linhas 28-30: na frase “O enfrentamento à mortalidade neonatal precisa ser considerado no planejamento e gestão em saúde (?) uma linha de cuidado que perpassa pela gestação, parto, nascimento e recém-nascido” parece faltar um conector entre as duas sentenças principais.

Resultados

Na Figura 1, parece haver mais um ponto após o ano 2020. Há ainda que se considerar o período da pandemia e a possível interferência nos resultados apresentados. Os dados da Tabela 1 poderiam ser incluídos como legenda da Figura 1, acrescentando o APC como caixa de texto no gráfico, pois se referem aos mesmos dados. Pelas novas normas da revista, sugere-se incluir as abreviaturas diretamente no título da ilustração. 

Disponibilidade de dados (página 8/18). Verificar concordância nominal - Ajustar para “os bancos de dados estão disponíveis...” indicando que os conteúdos estarão disponíveis no momento da publicação do artigo.

No formulário de concordância com a Ciência Aberta, porém, os autores indicam que “(x ) dados estão disponíveis sob demanda dos pareceristas”. Favor ajustar.

Discussão

Pelas instruções aos autores, orienta-se que as limitações do estudo sejam apresentadas no segundo parágrafo da discussão, para depois interpretar cada resultado relevante e discuti-lo à luz da literatura.

Página 5, linha 15: “Este aumento no estado da Bahia”, sugere-se indicar diretamente “na Bahia”. O mesmo

ocorre na linha 46 e, na página 7, linhas 45 e 54.

Página 5, linhas 53-60: sugere-se rever a pontuação da sentença.

Página 6, linhas 9-16: não fica muito claro o ponto de vista dos autores, pois não é apresentada relação entre a gravidez única e o parto vaginal com as condições socioeconômicas desfavoráveis. Sugere-se detalhar. Na interpretação dos achados apresentada ao final da página 6 e início da página 7, linhas 3 - 8, sugere-se contextualizar o acesso a exames específicos e cuidados intensivos no âmbito estadual, se possível.

Nas linhas 55-57, no trecho “Necessita-se de uma articulação que envolva saúde, desenvolvimento social, educação, infraestrutura, dentre outros. Para isso, uma boa articulação entre união, estado e municípios se faz indispensável” sugere-se alterar a forma de escrita, evitando repetição de ideias (e da palavra “articulação”), assim melhorando a clareza da conclusão do estudo.

Recommendation: Major Revision

## Second round comments

Caros autores

Agradecemos pela apresentação da nova versão do manuscrito e parabenizamos novamente pelo trabalho. Cabem ainda pequenos ajustes no seu texto, antes de nossa decisão final, os quais estão detalhados a seguir:

Resumo

Na conclusão do resumo, ajustar a concordância em “O aumento de óbitos evitáveis enfatiza(m) a necessidade de ampliar e qualificar os serviços de saúde, por meio de políticas públicas que abordem as desigualdades regionais e fortaleçam a atenção integral à saúde materno-infantil”.

Métodos

Retirar do texto dos métodos, aspectos estatísticos, a última frase “Uma vez que este estudo foi realizado a partir de dados de domínio público (dados agregados), não foi necessária a aprovação por um Comitê de Ética em Pesquisa”.

Resultados

Na 1ª frase dos resultados, poderia ser incluída a taxa de mortalidade neonatal para o período completo, ainda que não se use o termo ´média´. “No período de 2010 a 2020, foram contabilizados 2.246.730 nascidos vivos e 26.661 óbitos neonatais no estado da Bahia, gerando uma taxa de ....”.

Na última frase dos resultados, faltou pontuação em “Nas causas “reduzíveis por adequada atenção ao recém-nascido” foi observada tendência decrescente apenas em infecções específicas do período neonatal (exceto síndrome da rubéola congênita e hepatite viral congênita) (X) as demais variáveis tiveram tendência estacionária (Tabela 4).”

Discussão

Sugere-se indicar na discussão (junto ao 5º parágrafo) que foram comparados os dados com e sem a inclusão de 2020 e não se observou diferenças nas tendências da mortalidade neonatal, neonatal precoce e tardia. Ainda que não tenha sido objetivo do estudo calcular as taxas de mortalidade segundo as características sociodemográficas maternas, obstétricas e do recém-nascido, estas características influenciam nos dados apresentados e na interpretação destes, tanto que compõe um parágrafo e uma tabela de resultados. 

Dessa forma, importante mencionar na discussão / limitação que o uso das proporções apresentadas sofre influência da completitude dos dados, como destacado no texto “mais de 20% desses dados estavam como ignorados”. Cabe também destacar que as maiores proporções de óbitos sofrem influência das proporções de nascimentos daquela categoria. Assim, no 7º paragrafo da discussão, quando se apresenta uma contradição entre a literatura e os dados do estudo “No âmbito das características gestacionais, observou-se maior frequência de óbitos entre aqueles nascidos de gravidez única e via de parto vaginal, resultado semelhante ao encontrado em estudo que investigou a mortalidade perinatal em uma capital do Nordeste do Brasil (24). Contudo, a literatura aponta a gravidez múltipla e a cesariana como fatores de risco para a mortalidade neonatal (5)”, faz-se necessário acrescentar como possível justificativa para a discrepância as diferenças no método de apresentação dos dados, pois a proporção de óbitos é maior nas categorias mais frequentes de nascimento (denominador), enquanto a taxa relativiza os dados de acordo com a representatividade de cada categoria populacional.

Recommendation: Minor revision

## Third round comments

Caros autores

Reavaliamos atentamente a sua nova versão do manuscrito, e cabem ainda alguns esclarecimentos e ajustes, detalhados a seguir:

1. Na conclusão do resumo, o texto apresentado parece um pouco contraditório, sugere-se rever para apontar uma contradição e argumentar em relação a ela: “Apesar da queda na taxa de mortalidade neonatal... houve aumento de óbitos evitáveis”? Mesmo indicando avanços na assistência pré-natal e na atenção ao parto, é preciso qualificar os serviços de saúde? O aumento de óbitos evitáveis não foi geral, mas para grupos de causas específicos, segundo dados da Tabela 4. Salientar os fatores relacionados a atenção à mulher na gestação, por exemplo.

2. Disponibilidade de dados: Nesse item, deve-se indicar o acesso aos dados específicos do artigo - já trabalhados, indicadores calculados, indicando inclusive uma possível relação entre SIM - SINASC - e não os dados brutos acessados via DATASUS, cujo acesso é público. Indica-se que “Os dados foram extraídos do Sistema de Informações sobre Mortalidade e do Sistema de Informações sobre Nascidos Vivos o uso do SIM e SINASC” neste item e nos métodos, no entanto, em fonte de dados e mensuração, não há detalhamento de quais variáveis foram acessadas em cada um dos sistemas, ou se houve algum tipo de vinculação (linkage) de dados. Por favor rever e indicar adequadamente nos dois itens (métodos- fonte de dados e mensuração) e Disponibilidade de dados.

3. Na discussão, 7º parágrafo, o texto ainda está confuso. Recomenda-se melhorar a argumentação e salientar o uso das proporções, que sofre influência dos totais populacionais, diferenciando em relação ao uso de taxas. Por exemplo, por que quando analisado a taxa de mortalidade neonatal esse resultado é diferente?

Deve-se argumentar a luz das evidências da literatura.

Recommendation: Minor revision

